# Craniofacial fibrous dysplasia with cystic degeneration – A diagnostic challenge

**DOI:** 10.4317/jced.60736

**Published:** 2023-09-01

**Authors:** Fábio-Abreu Alves, Bruno-Henrique-Figueiredo Matos, Oslei-Paes de Almeida, Giovanna-Lopes Carvalho

**Affiliations:** 1 DDS, PhD - Department of Stomatology, A.C.Camargo Cancer Center, São Paulo, Brazil; 2 DDS, PhD - Lavras University Center, Lavras, Brazil; 3 DDS, PhD - Department of Oral Diagnosis, Piracicaba Dental School, University of Campinas, Piracicaba, Brazil; 4 DDS, PhD student - Department of Stomatology, School of Dentistry, University of São Paulo, São Paulo, Brazil

## Abstract

Benign fibro-osseous lesions with cystic degenerations have been scarcely reported in craniofacial bones and its unusual characteristics pose a diagnostic challenge. Here, we report a case of craniofacial fibrous dysplasia presenting a large cystic degeneration. A 55-year-old woman had a history of pain, slight asymmetry on the zygomatic region and ocular pressure. Computed tomography revealed on the right side, multiple craniofacial bones showing a ground glass aspect, associated with an extensive hypodense, unilocular, well circumscribed lesion in the maxilla, and smaller lesions in the sphenoid bone. After a surgical procedure performed in another service, there was a complete improvement in symptoms, and after 1 year, the patient remains stable, with no changes. In the literature review, thirty-three reported cases of the same association in the craniofacial region were found. The main symptoms were sudden increase in the lesion and pain, and the indication of intervention in cystic lesions was only indicated in symptomatic cases or functional deficit. The knowledge of the possibility of the association of benign fibro-osseous lesions and cystic degenerations in craniofacial bones is essential to perform a correct diagnosis and treatment for these patients, consequently avoiding unnecessary procedures.

** Key words:**Craniofacial fibrous dysplasia, Fibrous dysplasia, Benign fibro-osseous lesions, Cystic degeneration.

## Introduction

Benign fibro-osseous lesions constitute a group of diseases, in which healthy bone is replaced by fibrous tissue with foci of bone neoformation. The 3 most common lesions affecting craniofacial bones are fibrous dysplasia (FD), cemento-osseous dysplasia (COD), and ossifying fibroma (OF). Although, such diseases differ clinically and radiographically , they present similar histopathological features (1).

FD is caused by a post-zygotic mutation in the GNAS 1 gene that is linked to changes in osteoprogenitor cells, leading to abnormal bone formation. Monostotic FD affects only one bone and polyostotic several bones and may be associated with syndromes, such as McCune-Albright. The term craniofacial FD is used for FD involving multiple skull bones (1-3). There are few reports showing cystic degenerations in benign fibro-osseous lesions. These lesions include aneurysmatic bone cyst (ABC), simple bone cyst (SBC), and nonspecific cystic degeneration (CD) (4-7). Due the scarcity of data considering benign fibro-osseous lesions and cystic formations in craniofacial bones, the diagnosis may be a challenge for clinicians and radiologists. According to our knowledge, a total of 30 cases of FD in craniofacial bones presenting cystic formations have been reported in the English language literature (1980-2020), and here we present a new case of FD presenting nonspecific cystic degenerations.

## Case Report

**Figure 1:**
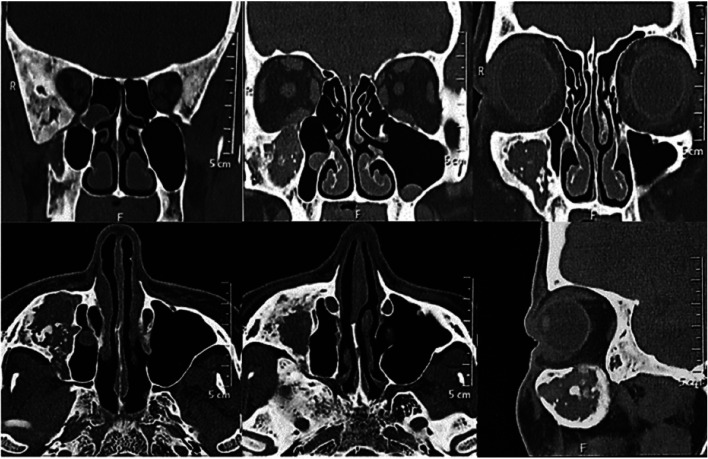
Computed tomography (CT) performed 6 years ago showed an extensive and expansive lesion with a mixed aspect such as “ground glass”. The lesion involved the maxilla, greater sphenoid wing, temporal, frontal, pterygoid process, floor and lateral wall of the orbit of the right side. It was also observed an extensive hypodense and unilocular area in the right maxilla, which caused a remodeling of the lateral and inferior walls of the ipsilateral maxillary sinus, decreasing its dimensions. Moreover, a cortical thickening of orbital floor and anterior wall of the maxillary sinus was observed. Similar lesions (in smaller size) were also observed in the sphenoid bone.

**Figure 2:**
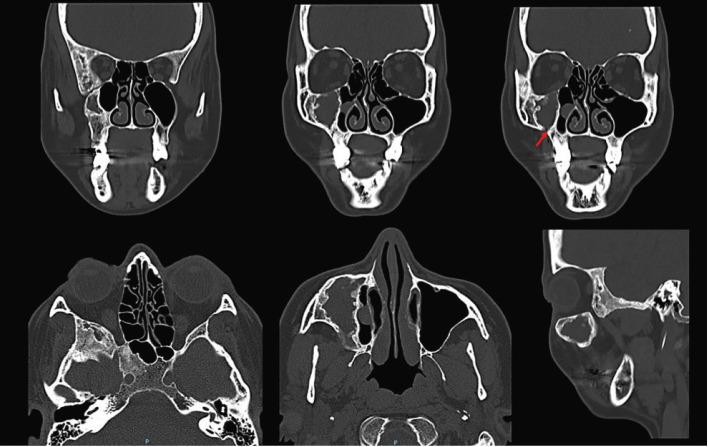
CT performed after the surgical procedure, there was no relevant alterations in the radiographic features when compared to the previous exams (Fig. 1), except for an area which corresponds to the surgical access (red arrow).

**Figure 3:**
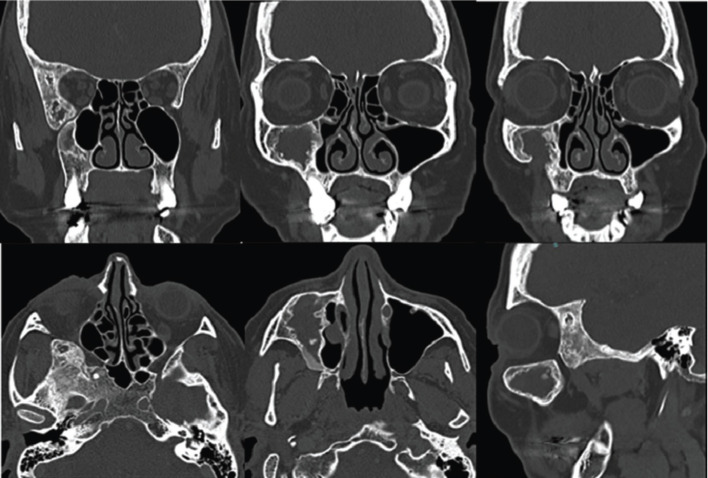
CT performed one year after the surgical procedure, the exam shows similar features.

A 55-year-old woman was referred to the Stomatology Department for evaluation of an injury on the right maxilla. During anamnesis, the patient reported to be treated and followed for 5 years by either otorhinolaryngologist and maxillofacial surgeon. She also had a history of pain and eye pressure, on the right side of the face, that ameliorated after a surgical procedure. In fact, the surgeon informed us that he found an empty cavity during the exploratory surgery. Due to doubts in relation to diagnosis, he referred the patient for our evaluation. On clinical examination, it was observed a slight swelling on the right side of the face. Computed tomography (CT) performed 6 years ago showed an extensive and expansive lesion with a mixed aspect mainly “ground glass”. The lesion involved the maxilla, greater sphenoid wing, temporal, frontal, pterygoid process, floor and lateral wall of the orbit, all on in the right side. It was also observed an extensive hypodense, unilocular, well circumscribed lesion in the maxilla. Such lesion caused a remodeling of the lateral and inferior walls of the ipsilateral maxillary sinus, decreasing its dimensions. Moreover, a cortical thickening of orbital floor and anterior wall of the maxillary sinus was observed. It is worth mentioning that similar lesions, but smaller, were also observed in the sphenoid bone (Fig. 1). In the CT after the surgical procedure, there was no relevant alterations in the radiographic features when compared to the previous exams, except the continuity solution in the maxilla, compatible with the surgical procedure performed (Fig. 2). According to both clinical and radiographic features, the diagnosis of craniofacial fibrous dysplasia associated with cystic degenerations was established. In the CT control after one year, there are no changes, the patient is asymptomatic (Fig. 3).

## Discussion

The association between craniofacial FD and cystic formations is uncommon, with few cases reported in the literature. The exact pathogenesis of this association is unknown. El Deeb (8) and Wojno & McCarthy (7) suggested that benign fibro-osseous lesions can lead to vascular and hemodynamic changes and deficient bone structural support favoring cystic formations. According to Ferreti (5) cystic lesions arise due to an intraosseous vascular defect, causing intramedullary hemorrhage. If direct communication occurs with the bleeding region, an aneurysmatic bone cyst is formed, but a complete interruption of blood supply leads to the formation of a simple bone cyst. A previous study of our group (9) reported a series of cases of the association between SBC and benign fibro-osseous lesions, and one particular associated with FD in the mandible. It is accepted that the mandible is more commonly affected due to less vascularized bone, facilitating osteocyte death and cystic formations. In a retrospective study conducted at the Chang Gung Craniofacial Center, 9 out of 113 cases of craniofacial FD were associated with cystic degeneration, more commonly in the sphenoid bone. The authors did not find a clear predisposing evidence for cystic degenerations (4) occur.

We found a total of 33 cases of FD presenting with cystic formations (SBC, ABC and CD) in craniofacial bones in the English literature (Table 1, 1 cont.). The average age was 21 years (ranging from neonate to 55 years). The main signs/symptoms were a sudden increase in volume, pain, and visual changes. Regarding radiographic characteristics, this association shows typical features of FD with a cystic component, which can be multiloculated (ABC) or uniloculated (SBC). In our case, an extensive uniloculated radiographic image was observed in the maxilla, and smaller areas in sphenoid associated with typical craniofacial FD. It is noteworthy that our patient has been followed up for 6 years with periodical tomography, showing only discrete alterations in the radiographic exams (Figs. 1,3). Such association, FD and CD, affecting the maxilla has been reported in only two cases in English literature (4,10), and other 2 cases of FD and ABC (4,7,11-13). There is no reported case in the literature of an association between FD and SBC affecting the maxilla, in our knowledge.

**Table 1:**
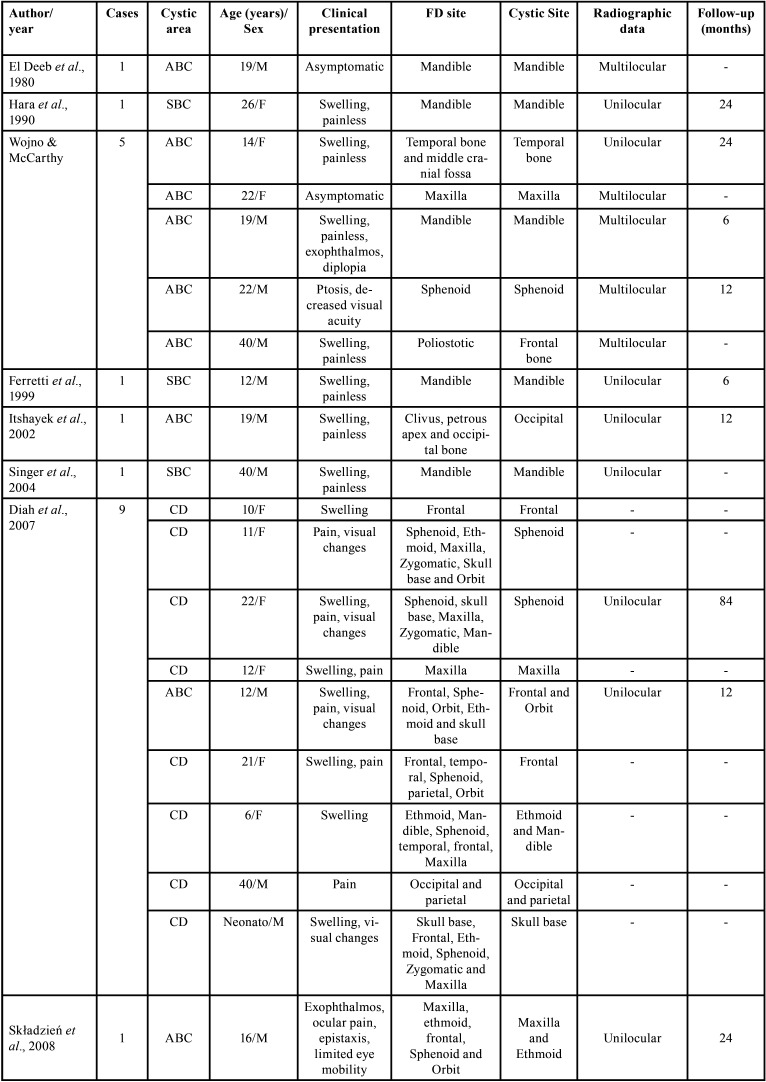
Cases reported in the literature of fibrous dysplasia in craniofacial bones associated with cystic formations.

**Table 1 cont.**
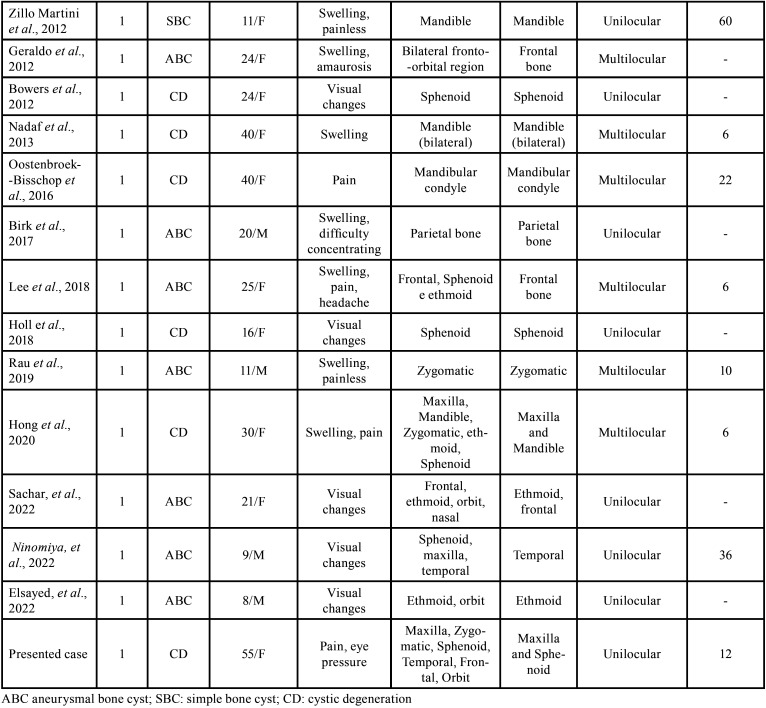
Cases reported in the literature of fibrous dysplasia in craniofacial bones associated with cystic formations.

Regarding treatment, it is suggested that symptomatic patients and/or presenting functional deficit, the cystic lesions should be promptly treated, otherwise the patient should be only followed up. Hong *et al*. (10) reported a case of a patient with FD and cystic degeneration affecting both mandible and maxilla. The patient had mandible pain, which was ameliorated after cystic assessment, with no further interventions. In our case, the surgical intervention, with cystic decompression, caused improvement of the ocular pressure and pain. The patient remains sTable, without symptoms. Current CT has showed no further alterations in the lesions. The patient is under follow-up and no surgical interventions has been indicated.

In conclusion, the present study described a rare case of craniofacial fibrous dysplasia with cystic degeneration. To our knowledge, this is the third case of such association in the maxilla. The knowledge of the association of benign fibro-osseous lesions and cystic degenerations in craniofacial bones is essential, considering that in most of the cases the treatment is expectant or performing only cystic decompression, and large resection must be avoided.

## References

[1] Nelson BL, Phillips BJ (2019). Benign Fibro-Osseous Lesions of the Head and Neck. Head Neck Pathol.

[2] Ahmad M, Gaalaas L (2018). Fibro-Osseous and Other Lesions of Bone in the Jaws. Radiol Clin North Am.

[3] Javaid MK, Boyce A, Appelman-Dijkstra N, Ong J, Defabianis P, Offiah A (2019). Best practice management guidelines for fibrous dysplasia/McCune-Albright syndrome: a consensus statement from the FD/MAS international consortium. Orphanet J Rare Dis.

[4] Diah E, Morris DE, Lo LJ, Chen YR (2007). Cyst degeneration in craniofacial fibrous dysplasia: Clinical presentation and management. J Neurosurg.

[5] Ferretti C, Coleman H, Altini M (1999). Cystic degeneration in fibrous dysplasia of the jaws: A case report. Oral Surg Oral Med Oral Pathol Oral Radiol Endod.

[6] Sharma G, Kamboj M, Narwal A, Devi A, Singh V, Gupta A (2019). A Rare Case of Ossifying Fibroma with Cystic Degeneration: Diagnostic Challenge with Literature Review. Indian J Otolaryngol Head Neck Surg.

[7] Wojno KJ, McCarthy EF (1994). Fibro-osseous lesions of the face and skull with aneurysmal bone cyst formation. Skeletal Radiol.

[8] El Deeb M, Sedano HO, Waite DE (1980). Aneurysmal bone cyst of the jaws - Report of a case associated with fibrous dysplasia and review of the literature. Int J Oral Surg.

[9] Zillo Martini M, Caroli Rocha A, Lemos CA, Abreu Alves F (2010). Fibro-osseous lesions associated with simple bone cysts: three case reports and review of the literature.

[10] Hong I, Kang DC, Leem DH, Baek JA, Ko SO (2020). An unusual presentation of non-specific cystic degeneration of craniofacial fibrous dysplasia: a case report and review of literature. Maxillofac Plast Reconstr Surg.

[11] Lee HS, Koh YC, Roh HG, Park HK, Kim SY (2018). Secondary Aneurysmal Bone Cyst in a Craniofacial Fibrous Dysplasia: Case Report. Brain Tumor Res Treat.

[12] Rau LH, Reinheimer A, Meurer MI, Marodin AL, Espezim CS, Klüppel LE (2019). Fibrous dysplasia with secondary aneurysmal bone cyst-a rare case report and literature review. Oral Maxillofac Surg.

[13] Składzień J, Oleś K, Zagólski O, Moskała M, Sztuka M, Strȩk P (2008). A giant cranial aneurysmal bone cyst associated with fibrous dysplasia. B-ENT.

[14] Hara H, Ohishi M, Higuchi Y (1990). Fibrous dysplasia of the mandible associated with large solitary bone cyst. J Oral Maxillofac Surg.

[15] Itshayek E, Spector S, Gomori M, Segal R, Al ET (2002). Fibrous dysplasia in combination with aneurysmal bone cyst of the occipital bone and the clivus: case report and review of the literature. Neurosurgery.

[16] Singer SR, Mupparapu M, Rinaggio J (2004). Clinical and radiographic features of chronic monostotic fibrous dysplasia of the mandible. J Can Dent Assoc (Tor).

[17] Geraldo A, Santos D, Mendes C, Tavares J, Sousa R, Campos A (2012). Lesões Fibro-ósseas Craniofaciais Benignas com Quistos Ósseos Aneurismáticos Associados. Acta Med Port.

[18] Bowers C, Altay T, Shah L, Couldwell WT (2012). Pregnancy-induced cystic degeneration of fibrous dysplasia. Can J Neurol Sci.

[19] Nadaf A, Radhika M, Paremala K, Srinath N (2013). Monostostic fibrous dysplasia with nonspecific cystic degeneration: A case report and review of literature. J Oral Maxillofac Pathol.

[20] Oostenbroek-Bisschop J, Verweij J, van Merkesteyn JP (2016). Custom Made Replacement of the Mandibular Condyle in a Case of Fibrous Dysplasia with Cystic Degeneration; A Case Report. Dent J.

[21] Birk H, Winkler EA, Bonney PA, Berger MS, McDermott MW (2017). Calvarial aneurysmal bone cyst associated with fibrous dysplasia: Case report and literature review. Interdiscip Neurosurg Adv Tech Case Manag.

[22] Holl DC, Hardillo JAU, Dammers R, van der Schroeff MP, van der Lugt A (2018). Cystic Degeneration of Craniofacial Fibrous Dysplasia. World Neurosurg [Internet].

[23] Sachar C, Agarwal A, Syed A (2022). Bony Cyst in a Ground Glass Matrix: A Rare Case Report of Craniofacial Fibrous Dysplasia with Secondary Aneurysmal Bone Cyst. Indian J Radiol Imaging.

[24] Ninomiya H, Ozeki M, Nozawa A, Yasue S, Endo S, Inuzuka M (2022). A rare pediatric case of McCune-Albright syndrome with acute visual disturbance. Medicine (Baltimore).

[25] Elsayed AA, Mohamed RMH, Devine JC, Wasserberg J, Elbadawey MR, Abdelsamad HSS (2022). Aneurysmal bone cyst on top of fibro-osseous lesion of the ethmoid sinus with orbital and intracranial extension in a child. BJR|case reports.

